# Crosslinking of Polylactide by High Energy Irradiation and Photo-Curing

**DOI:** 10.3390/molecules25214919

**Published:** 2020-10-23

**Authors:** Melania Bednarek, Katarina Borska, Przemysław Kubisa

**Affiliations:** 1 Centre of Molecular and Macromolecular Studies, Polish Academy of Sciences, Sienkiewicza 112, 90-362 Lodz, Poland; katarina.borska@savba.sk (K.B.); pkubisa@cbmm.lodz.pl (P.K.); 2 Polymer Institute, Slovak Academy of Sciences, Dubravska Cesta 9, 845 41 Bratislava, Slovakia

**Keywords:** polylactide, poly(lactic acid), crosslinking, photo-crosslinking, irradiation, electron-beam, gamma rays

## Abstract

Polylactide (PLA) is presently the most studied bioderived polymer because, in addition to its established position as a material for biomedical applications, it can replace mass production plastics from petroleum. However, some drawbacks of polylactide such as insufficient mechanical properties at a higher temperature and poor shape stability have to be overcome. One of the methods of mechanical and thermal properties modification is crosslinking which can be achieved by different approaches, both at the stage of PLA-based materials synthesis and by physical modification of neat polylactide. This review covers PLA crosslinking by applying different types of irradiation, i.e., high energy electron beam or gamma irradiation and UV light which enables curing at mild conditions. In the last section, selected examples of biomedical applications as well as applications for packaging and daily-use items are presented in order to visualize how a variety of materials can be obtained using specific methods.

## 1. Introduction

Polylactide/poly(lactic acid) (PLA) is a biodegradable and biobased aliphatic polyester derived from renewable sources such as corn, potato, and sugar cane. Due to biodegradability and biocompatibility (PLA is approved by US Food and Drug Administration for contact with human cells) the early applications of polylactide (or its copolymers with polyglycolides) involved surgical sutures, implants or drug formulations [1,2,3,4,5]. The use of PLA was initially limited to these biomedical applications due to its high cost and low availability.

Biodegradability is considered as one of the major advantages of polylactide, thus, PLA materials (apart from biomedical applications) have been also used for the production of short-use items and packaging [1]. More recently, however, there have been some concerns related to the environmental impact of the utilization of biodegradable polymers as the dumping of biobased waste in landfills contributes to global warming and leachate [6,7]. Thus, in recent years the shift from degradability/“compostability” to “renewability” and an increasing interest in using PLA based products for long-term usage applications, even at the expense of reducing the biodegradability of the polymer may be observed [1]. It is expected that PLA will have broader applications in the medical and food industries, however, much has to be overcome to ensure actual sustainability, including enhancement of mechanical and thermal properties [8]. There are many efforts to improve the performance of PLA via different modification methods including copolymerization, blending, or crosslinking [9,10,11,12]. The design of novel structures such as networks based on polyesters offers a possibility for enhancing mechanical and thermal properties. The improvement of mechanical strength is needed for both medical devices and parts of daily-use items. However, when designing materials for these applications, an awareness that the crosslinking process leads not only to the enhancement of polymer toughness (usually with the improvement of thermal properties) but also to the modification of other properties as degradability, solubility, gas permeability, and so on must be maintained. Basically, crosslinking leads to a decrease in degradability and this may be a negative feature for materials designed for biomedical applications (implants and drug delivery systems). However, in some cases, longer times of implant destruction or longer delivery of the pharmaceutical agent could be required. Thus, very detailed studies of crosslinking of biobased and biocompatible materials include investigation of their degradation in different environments and their biocompatibility. The balance between mechanical strength and degradability of materials should be established at the stage of their design in order to fulfill the requirements of the target application.

Networks can be obtained through different approaches. Applied methods are usually classified into several main groups, that is chemical crosslinking and crosslinking by exposure to low- energy light or ionizing radiation. Chemical crosslinking is one of the largest groups because, among others, this group includes all methods based on radical crosslinking induced by peroxides [13].

Both high-energy radiation crosslinking and photo-crosslinking are relatively easy methods in comparison with chemical crosslinking, however, some advantages and weakness of each of them are known. Photo-crosslinking requires the presence of reactive -unsaturated groups in polymer chains (functionalization of synthesized polymers) and the presence of photo-initiators but may be accomplished under mild conditions (room temperature) for different materials (solid, liquid, containing different encapsulated compounds including sensitive proteins, etc.) [14]. High energy radiation, such as gamma and electron beam radiation has been applied for various treatments of polymers, and also crosslinking [15,16]. Crosslinking by irradiation may be performed on pristine polymer without the necessity of functionalization because radicals may be generated directly on polymer chains. However, it is accompanied or even dominated by chain scission. Thus, even in this case using additives (crosslinking monomers) is preferred.

This review does not include chemical crosslinking which has been broadly covered by other articles [13,17]. Instead, the present state of the art concerning the formation of crosslinked PLA-based materials by photo-crosslinking and high energy irradiation is presented. Totally different materials are obtained depending mainly on crosslinking density which, in turn, depends on the radiation type, its dose, conditions, and on PLA-based polymer (a composition and an architecture of this (co)polymer have an independent contribution to the overall properties).

## 2. PLA Crosslinking by Electron Beam or Gamma Irradiation

Ionizing radiation has been well known as a very convenient tool for the modification of polymeric materials through crosslinking, grafting, and degradation. PLA however, undergoes predominantly degradation under the influence of ionizing radiation. Thus, the mechanical and physical properties of polymers exposed to gamma rays or an electron beam, decrease due to the reduction in molecular weight.

**Figure 1:**
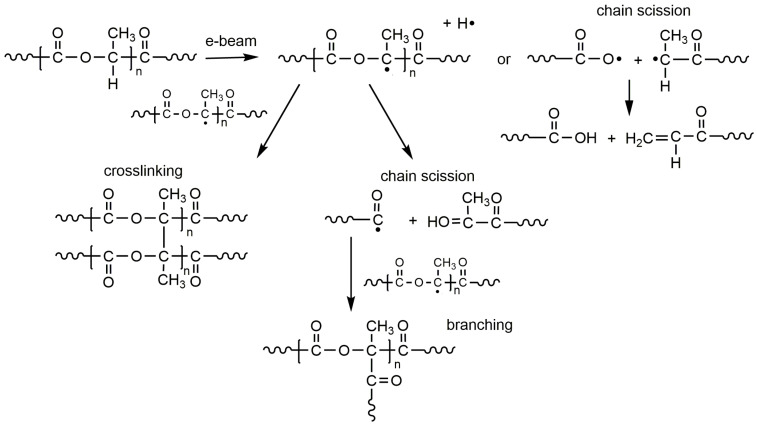
Possible processes involving radicals on polylactide (PLA) chains formed by irradiation with an electron beam (on the basis of Reference [27]).

Beginning with the advent of research related to the development of polyester materials for the production of implants, surgical sutures, and drug delivery systems, high energy irradiation, mainly gamma but also electron irradiation has been used for sterilization [18,19,20,21]. It has been found that, depending on the chemical structure of the polymers, the absorbed dose, the dose rate, and the temperature of irradiation, various reactions involving radicals generated along the polymer chain proceeded such as chain scission and crosslinking reactions which were often accompanied by the evolution of gaseous products [15,22,23,24,25,26,27]. Radical processes which may proceed during high energy irradiation [27] (see Figure 1) have been proposed.

Reactions under high energy radiation leading to the destruction of the PLA chain were useful for the controlled PLA degradation [19,28,29].

**Figure 2:**
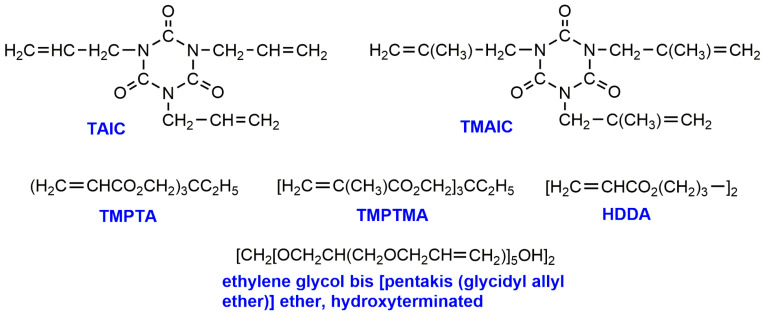
Multifunctional crosslinking agents applied in radiation-induced crosslinking [30]. (Adapted with permission from Elsevier, 2005).

Many authors studied gamma radiation-induced changes (decreases) in the enthalpy of melting and cold crystallization, the degree of crystallinity, the glass transition temperature, and the thermal stability of polylactides [24,27,29]. It is obvious that the extent of the drop of mentioned parameters increases with the increase of radiation dose. The chain scission of polymer chains under irradiation is accompanied by the crosslinking process especially when higher doses are applied [20]. The polymer crosslinking may dominate at appropriate conditions (below doses of 250 kGy mainly chain scission proceeds [19]) and can be done on purpose. Biodegradable polymers that could be crosslinked by irradiation would be valuable not only in the medical field but for other industrial applications as well. Introducing crosslinking into biodegradable polymers, should result in an enhancement of mechanical properties and delayed hydrolysis of the polymer. To overcome the effect of significant molecular weight decrease, the curing of PLA is frequently performed in the presence of polyfunctional monomers. In irradiation-induced crosslinking such compounds as triallyl isocyanurate (TAIC), trimethallyl isocyanurate (TMAIC) trimethylolpropane triacrylate (TMPTA), trimethylolpropane trimethacrylate (TMPTMA), 1,6-hexanediol diacrylate (HDDA) and ethylene glycol bis (pentakis (glycidyl allyl ether)) ether were applied [30]. The structures of these monomers are shown in Figure 2.

Polyfunctional monomers have been applied predominantly in electron beam-induced crosslinking [27,30,31,32,33,34,35] although they have been also used for curing by γ-irradiation [36,37,38].

Mitomo and his group studied the effects of the type and the concentration of polyfunctional monomer as well as parameters of the irradiation with electron beam (the irradiation dose, temperature) on the crosslinking of poly(l-lactide) (PLLA) or mixture of poly(l-lactide)/poly(d-lactide) (PLLA/PDLA), the thermal properties, and the biodegradation of obtained crosslinked polymers [30,31]. It was found that the most optimal conditions to introduce crosslinking were around 3% of TAIC and the irradiation dose of 30–50 kGy [30,33]. The crosslinked PLA films had much improved heat stability and mechanical properties. The resultant properties of PLA samples were governed by crosslinking density which depended on the structure and length of PLA chains and on the radiation dose. The crosslinked PLA became harder and more brittle at low temperatures, but was rubbery, soft, and stable at higher temperatures, even over T_m_. The degradation of irradiated – crosslinked PLA samples was considerably retarded.

Some authors studied the results of the crosslinking of PLA blends and composites with other materials (PCL, poly(butylene adipate-co-terephthalate) (PLA/PBAT), flax fibers, montmorillonite, and others) under electron beam irradiation [34,39,40,41,42,43]. The physical properties, apart from crosslinking conditions, depended strongly on the blend composition. It was stated that TAIC was an efficient agent that hindered the phase separation and linked macromolecules of both the same and the different polymers. On the other hand, the addition of crosslinking monomers (TAIC) was claimed to hamper polyester degradation [41].

Various additives have been also added for PLA crosslinking by γ-rays. For example, blends of PLA with flax fibers were subjected to γ-irradiation [37], octavinyl-POSS (octavinyl polyhedral oligomeric silsesquioxane) was used as an additional crosslinking agent [44], or PLA was blended with epoxy-functional acrylic oligomer as a chain extender in order to receive higher M_n_ and improved properties of PLA-based material [38].

These composite materials have been prepared with the support of ionizing radiation in order to modify the PLA properties, mainly with the aim of their use in packaging and in the production of consumer goods. The radiation-induced radicals on different components of blend/composite are able to react forming linkages between separate phases leading to the increase of the compatibility between components or to the formation of a multicomponent network. Some examples of the preparation of composite materials for daily use applications are shown in Section 4.

The attempted crosslinking of different polylactide-based polymers by using high-energy irradiation and different crosslinking agents is presented in Table 1.

## 3. Photo-Crosslinked PLA

Photo-initiated crosslinking has many advantages for biomedical applications because it allows fast crosslinking under mild reaction conditions without solvents. Radical crosslinking using peroxides is not appropriate for biomedical applications because of the toxicity of the decomposition products of peroxides and not defined degradation products. Electron beam and γ-irradiation crosslinking require a large amount of radiation energy and the presence of a crosslinking agent to get the advantage of crosslinking over chain scission. Photo-crosslinking provides significant advantages over these two approaches, such as ease of use, safety, especially in connection to living systems, and low cost [45], although it may also be accompanied by other processes leading to polymer degradation. UV was also intentionally used to induce polylactide degradation (through, e.g., radiolysis or photo-oxidation) [46].

Polylactide intended for crosslinking is first functionalized at the chain ends with double bonds and then subjected to UV or visible light/laser irradiation which induces radical polymerization. To initiate radical polymerization photoactive additives are added such as substituted phenylacetophenones (irgacures) or camphorquinone. In comparison with peroxide-induced crosslinking, the photo-crosslinking can be accomplished at low temperatures [47].

As it was mentioned PLA-based materials which were photo-crosslinked were designed for medical applications (tissue scaffolds or drug carriers), thus, this crosslinking method concerns mostly lactide copolymers i.e., PLA/ polyethylene glycol (PEG), poly(tetramethylene oxide) (PTMO), poly(ε-caprolactone) (PCL), polyglycolide (PGA), poly(trimethylene carbonate) (TMC) copolymers [46,47]. These were ABA type copolymers or statistical copolymers obtained by tin octanoate catalyzed ring-opening (co)polymerization of lactide initiated by polyether diol (ABA block copolymers), alternatively, by low molecular diols or multifunctional alcohols in the case of the statistical or star-shape copolymer. Obtained difunctional or multifunctional –OH terminated polylactides were functionalized by esterification, usually with (meth)acryloyl chloride. Low molecular weight polymers (oligomers) were mixed with photoinitiator and, often in molds, were exposed to UV lamp irradiation.

Based on acrylated PDLLA-PEG-PDLLA copolymers or functionalized with fumarate groups, upon crosslinking, either hydrolyzable gels [48,49] or tissue scaffolds with controlled macroscopic architecture, potentially for bone regeneration [50,51] were prepared. Water-soluble PEG/PLA copolymers consisted of PEG fragment with M_n_ = 1000–10,000 and the attached 2–40 LA units [48]. The authors claim that obtained nontoxic macromers could be photo-polymerized in vivo in direct contact with tissue. Prepared by another group, hydrogels (with about 2–4 LA units attached to PEG with M_n_ = 4000) were applied for encapsulation of model proteins [49].

Mechanical properties of networks for scaffold were highly dependent on the number of lactic acid and ethylene glycol units in the oligomer backbone ranging from 2–8 EG units and 6-10 LA units [52]. Also, hydrophobicity/hydrophilicity balance varied with copolymer compositions what was important with regard to polymer degradation and cell attachment. In both studies, the complete degradation of networks to water-soluble products was performed in physiological conditions.

The authors of another work [53] prepared copolymers consisting of polyethers such as PEG, poly(propylene glycol) (PPG) or poly(tetramethylene glycol) (PTMG) and 7–65 wt % of d,l-lactide units which, after functionalization introducing acrylate end groups, were subjected to UV irradiation. Photo-polymerization resulted in the network with gel content equal to 78% for copolymers with long PEG chain (M_n_ = 10,000) and over 97% for PEG and PPG with M_n_ around 400. Hydrophilic PEG-based networks rapidly degraded into completely water-soluble products within 1 day, while the degradation times of the more hydrophobic PPG and PTMG-based networks varied from 1 to 7 days. Obtained materials can potentially be used as biodegradable lubricants for coating various medical products.

In several articles, the syntheses of PLA-based copolymers by copolymerization of d,l-lactide with ε-caprolactone (CL), or L-lactide with ε-caprolactone and glycolide (GL) initiated with diethylene glycol or tetra(ethylene glycol), followed by the end-(meth)acrylation and crosslinking were described [51,54]. In the case of the first cited work PLA/PCL copolymers with M_n_ in the range 1500–2400 resulted in a rather dense network after crosslinking while in a second work, M_n_ of PLA/PCL/PGA terpolymers was in the range 1800–10,200 giving networks with varied mechanical properties depending on copolymer composition and molecular weight. Degradation of all networks has been studied as well as their biocompatibility, both in respect to their potential application in tissue engineering. Copolymerization of d,l-lactide with glycolide was also initiated by PEG (M_n_ = 1500) leading to PLGA–PEG–PLGA copolymers which were further functionalized with itaconic anhydride [55]. Crosslinking by UV irradiation resulted in hydrogels which, according to the authors, could be used in moist wound healing or as carriers for controlled drug release.

For tissue elastic implants, materials from PDLLA/1,3-trimethylenecarbonate (TMC) copolymers have been synthesized by UV coupling of linear macromers with methacrylate groups and relatively high molecular weights (~30,000) [56]. Obtained networks with tunable thermal and mechanical properties depending on DLLA to TMC ratio could be used as implantable devices having different geometries as well as porous scaffolds with shape-memory properties.

Many authors used star-shaped polylactides and lactide copolymers instead of linear ones for the photo-crosslinking. Starting their works with homopolymers, Grijpma group prepared star-shaped poly(d,l-lactide) oligomers with 3 and 6 arms, with arm molecular weight in the range 200–5700 [57], which, after functionalization with methacryloyl chloride, were diluted with ethyl lactate and subjected to photo-crosslinking. Networks prepared from macromers of which the molecular weight per arm was 600 or higher had good mechanical properties, similar to linear high molecular weight poly(d,l-lactide). Films and porous scaffolds with gyroid architecture have been prepared by stereolithography, using a liquid resin based on a 2-arm PDLLA macromer and ethyl lactate. It appeared that pre-osteoblasts showed good adherence to these photo-crosslinked networks. The same group prepared 3-arm copolymers of d,l-lactide with ε-caprolactone and 1,3-trimethylenecarbonate with M_n_ = 3100–4000 which subsequently were functionalized with fumarate groups [58]. UV-initiated polymerization proved the sufficient reactivity of these groups and resulted in networks with high gel content (up to 96%) which physical properties varied depending on the composition, and molecular weight of the oligomeric precursors.

Other authors prepared 4-arm PDLLA/PCL copolymers functionalized with (meth)acrylate groups [59,60]. One of these works concerned the investigation of thermal properties of prepared thermoresponsive membranes from prepolymers with M_n_ in the range 3200–12,000, designed for drug delivery [60]. The other was focused on different techniques of resistor preparation to achieve shape accuracy and edge sharpness of samples prepared from crosslinked PLLA/PCL stars with short arm length, i.e., ~2500 [59].

Significant achievements in the field of synthesis of photo-crosslinked materials based on polylactide, designed for biomedical applications has a group of Amsden [61]. They worked on bioelastomers which could be used for the production of tissue scaffolds and implantable devices for drug delivery. For this reason, copolymers of lactide with such comonomers as ε-caprolactone and trimethylene carbonate (or substituted carbonate) introducing flexibility were prepared. Similarly as it was in the study by other researches, star 3-arm d,l-LA (co)polymers, or occasionally linear oligomers with M_n_ usually in the range 1000–5000 were functionalized with acrylate groups. Alternatively, acrylate groups were introduced as side groups by copolymerization of lactide with cyclic carbonate substituted with these groups [62,63]. Functionalized prepolymers were crosslinked in the presence of photo-active compounds and sometimes together with co-crosslinkers as, e.g., poly(ethylene glycol) diacrylate [64,65,66,67]. Obtained networks were studied concerning for their mechanical properties, degradation, and biocompatibility. A large part of the works concerned the study of the encapsulation of biologically active compounds and their release. Specific properties achieved in particular studies described by group of Amsden in numerous articles are shown in Table 2 (at the end of this section). 

A similar approach as described above has been also used by other authors. Thus, 4-arm star PDLLA oligomers (containing ethylene glycol units in the initiator fragment), of different M_n_ (1500–9500) with either methacrylated or urethane methacrylated end groups have been synthesized and photochemically crosslinked [68]. High gel content networks (90–99%) had T_g_ strongly dependent on prepolymer molar mass. Mechanical properties depended on both the type of introduced end groups of prepolymer (methacrylate or urethane methacrylate) and molar mass [68]. 

Crosslinking of methacrylate-terminated linear d,l-lactide oligomers with M_n_ around 1300 has been also applied for the preparation of potential composite resin for stereolithography [69]. To enhance crosslinking, triethylene glycol dimethacrylate (TEGDMA) as reactive diluent has been added in the amount of 30% and 50%. PLAs together with TEGDMA was blended with hydroxyapatite (HA) in the amount of 20%, 30%, 40% and 60% to prepare composites that were next photopolymerized in the presence of photoinitiator giving products with gel content up to ~100 %. Analysis of the thermal properties of crosslinked composites showed that T_g_ significantly shifted to a higher temperature when HA was incorporated. It indicated the interaction between HA particles and PLA matrix, leading to a mobility restriction of the polymeric chains. The addition of HA also affected the thermal stability, as known from the thermogravimetric analysis—the shift to higher temperature was observed for crosslinked PLA containing HA. Degradation of composites has been investigated as well as changes in thermal and mechanical properties during degradation. Additionally, the cytocompatibility of cells in contact with composites with different HA contents during degradation has been studied. Lower cytotoxicity of degradation products was observed for a sample with a higher content of HA. As a conclusion, the authors claim, that materials showed their potential in a stereolithographic fabrication of bone implants.

Functionalized with (meth)acrylate groups star low molecular weight polylactides have been used for stereolithography also by other groups where two-photon polymerization (2PP) technique was applied for crosslinking [70,71,72,73,74,75]. Star-shaped methacrylate-terminated oligo(d,l-lactide)s with M_n_ = 2800 were prepared, and it was demonstrated that oligomer synthesis and their functionalization can be carried out in the same reactor [71]. Subsequently, 2PP technique was used to prepare hexagonal porous scaffold with 3D structures in the presence of photoinitiator. These fabricated scaffolds were shown as a beneficial microenvironment for osteogenesis and bone regeneration in vitro and in vivo. Similarly, fabricated scaffolds (2PP technique) were also used for supporting of Schwann cells growth and thus, as neural scaffolds in nerve repair [70]. Laser-induced crosslinked star-shaped methacrylate-terminated oligo(d,l-lactide)s (M_n_ = 2400) were used as a reinforcement of collagen materials [74,75]. The material exhibited improved resistance to biodegradation, while the direct multipotent stromal cell growth during their culture was observed. Reinforcement of collagen sponges resulted in near one order of magnitude increase of Young’s modulus without affecting of cytotoxicity and developed matrix provided cell adhesion and proliferation. Based on the results, the authors suggested this material for tissue engineering applications. 

All previous studies (above-mentioned works) were focused on crosslinking of PLA or PLA copolymers where curable groups were sited at the ends of polymer chains (end-functionalized polymers). In an alternative approach, poly(lactide-co-glycidyl methacrylate) (P(LA-co-GMA)) copolymer has been synthesized by ring-opening polymerization where curable C=C groups were placed in side-chains of the copolymer (pendant unsaturated groups) [76]. The copolymer was irradiated in the presence of an initiator and the influence of irradiation time, initiator concentration, as well as GMA content in polymer chain on crosslinking efficiency were followed by gel content measurement. Crosslinking led to the enhancement of mechanical and thermal properties and was dependent on the content of GMA units. In another study, P(LA-co-GMA) copolymer and its partly UV crosslinked counterpart were grafted with a pH-responsive polyacrylamide (PAAm), by UV-assisted reactions using acrylamide (AAm) and *N*,*N*′-methylene bisacrylamide monomers, and various photoinitiator systems [77]. These materials have the potential for use in biomedical and environmental applications due to their amphiphilic and pH-responsible properties.

**Figure 3:**
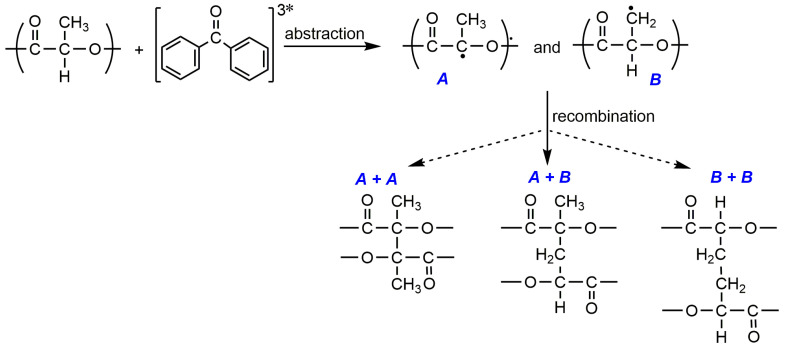
The proposed mechanism of photo-crosslinking of not functionalized PLA in the presence of benzophenone [78]. (Reproduced with permission from Wiley, 2013).

A different example of crosslinking is the application of high molecular weight/commercial PLLA for UV-induced crosslinking [78]. PLLA powders containing different concentrations of benzophenone (2–3.6 mol% per LA repeating unit) were hot-pressed at 190 °C and obtained films were continuously UV irradiated from both sides using different energy. Networks with gel content up to 98.5% have been prepared. By ^1^H and solid state ^13^C analyses of pristine PLA, the gel, and soluble fractions of the products, the authors suggested the mechanism of crosslinking which is presented in Figure 3. According to them, the photo-crosslinking may result from the recombination between primary and tertiary carbon radicals generated by the hydrogen abstraction from the PLA chain by the excited benzophenone.

DSC and XRD analyses indicated that prepared networks were partially crystalline up to 93% of gel content. T_g_ slightly increased because of the introduction of crosslinked structure in PLA, both T_c_ and T_m_ shifted to higher temperatures and finally disappeared with increasing gel fraction. The authors found that the crosslinks have been formed not only in the amorphous region but also in the crystalline region incorporating into the crosslinked network. The photoinitiator may penetrate into the crystalline region by the sublimation during the film formation. The photo-crosslinking improved mechanical properties by increasing both tensile strength and modulus by 70% with a little less decrease in elongation at break. Unexpectedly the toughness of the crosslinked PLA also increased by 22.5%. The authors named this type of crosslinking “crystal crosslinking” and claimed that described by them photo-crosslinking was more efficient compared with conventional amorphous crosslinking (much more significant improvements in thermal and mechanical properties).

**Figure 4:**
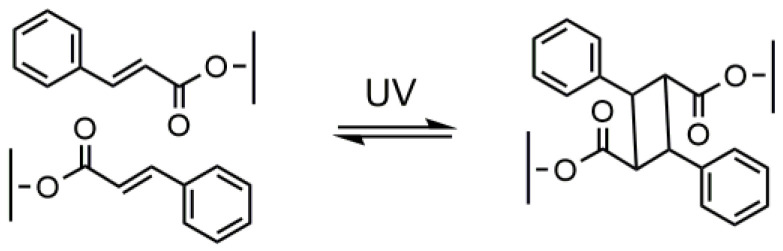
Cyclodimerization of cinnamoyl groups.

Photo-crosslinking has also been applied by several authors for the curing of PLA-based prepolymers using quite an alternative approach. In this approach, photochemically active groups were introduced not as photosensitive additives but directly into PLA chains. As photosensitive sites cinnamoyl groups were used which are able to dimerize upon UV light of appropriate wavelength according to the scheme shown in Figure 4, forming cyclobutane rings [79] and so bridges between PLA chains. 

**Figure 5:**
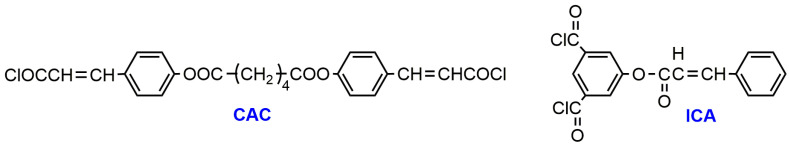
Compounds used for polycondensation with PLA diols.

The dimerization of cinnamoyl groups has been mainly used for the preparation of reversible networks (dimerization of cinnamoyl groups is reversible and cyclobutane ring undergoes cleavage under UV irradiation with another wavelength) [80,81], however, some authors didn’t study the mentioned reversibility. Thus, cinnamoyl groups were introduced into PLA chain by polycondensation [82,83] of PLLA diols (M_n_ in the range 1260–3010 or 2300–8900) with diacyl dichlorides containing these groups, i.e., with 5-cinnamoyloxyisophthalic acid (ICA) [82] or with diacyl chloride of 4,4′-(adipoyldioxy)dicinnamic acid (CAC) [83] (see Figure 5).

Polycondensates were subsequently crosslinked with the light of λ = 282 nm. Dimerization of cinnamoyl groups appeared effective and after 2 h of irradiation, approximately 90% of these groups disappeared [82]. The authors observed a decrease of crosslinking rate and the amount of formed gel with increasing M_n_ of ICA/PLLA copolymer, which they assigned to lower concentration of a photosensitive component in the sample. While for PLLA with M_n_ ~ 4000 the amount of gel content was 100%, for PLLA with M_n_ ~ 9000 it was only 50 % [82]. From a comparison of the crosslinking rate of copolymers ICA/PLLA and CAC/PLLA with the same M_n_ of PLLA-diols, the authors concluded that cinnamoyl moiety in the side-chain was more photoreactive than that in the main-chain [82]. In both cases, decrease in degradation rate was observed after crosslinking in comparison with un-crosslinked functionalized PLLA and neat telechelic PLLA. 

In another work, cinnamoyl moiety has been introduced into the PLLA side chain by copolymerization of lactide with cyclic carbonate monomer, i.e., 5-methyl-5-cinnamoyloxymethyl-1,3-dioxan-2-one (MC). Polymers of different ratios of MC/LA and M_n_ ranging from 12,900 to 65,100 were prepared. The crosslinking of the copolymer was followed by FTIR but no further properties of crosslinked material were discussed [84]. 

**Figure 6:**
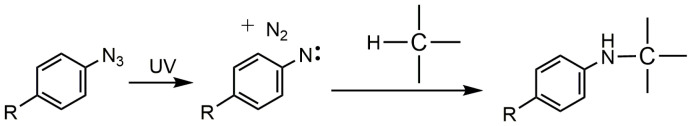
Mechanism of the formation of covalent bond between species bearing azide group and the compound with reactive hydrogen.

An original approach to PLA crosslinking has been recently presented by authors who applied multi(aryl azide) crosslinker for UV curing of PLA-Pluronic^®^ copolymer not containing unsaturated groups [85]. They adopted an earlier reported strategy of UV-induced polyester crosslinking [86] relying on the UV-activation of the aryl azide group to generate highly reactive nitrene species that can insert into carbon-hydrogen bonds of the polymer backbone, thereby leading to crosslinking via amine groups (see scheme in Figure 6).

Applying this elegant and straightforward strategy, using polymeric multi-azide crosslinker, which can be also used for the crosslinking of other not pre-functionalized polymers, the authors prepared degradable elastomers for soft tissue engineering. Interesting elastic scaffold prepared by electrospinning from above described materials will be shown in the last section of the review.

## 4. PLA-Based Materials by Photo- and High Energy Radiation Crosslinking

As mentioned in the previous sections, electron beam and gamma irradiations as well as UV light were predominantly used for crosslinking of PLA-based materials designed for biomedicine. However, in most articles concerning crosslinking by ionizing radiation only changes in properties of polylactide (co)polymers upon exposure to high energy radiation are discussed without indication of their specific medical application. On the other hand, some authors presented examples of daily use items prepared from gamma or electron beam irradiated PLAs.

**Figure 7:**
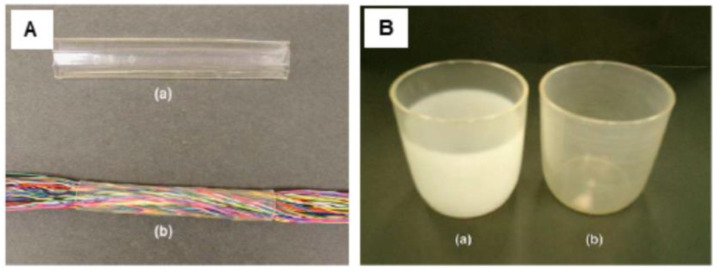
PLLA crosslinked by electron beam irradiation (50 kGy). (**A**) shrinkable tube (**a**); possible use (**b**). (**B**) Appearance of cups after using for hot water: (**a**) the unirradiated product, (**b**) the product crosslinked by irradiation [31]. (Adapted with permission from Elsevier, 2005).

Mitomo, who studied PLA crosslinking by electron beam irradiation for many years (the results are presented in Table 1 in Section 2) [29,30,31,32,33] demonstrated how irradiation improved the thermal stability of cups and plates prepared from poly(L-lactide) and how it enabled the preparation of heat-shrinkable tube which can be used as a cover for electric wire [31]. The tube was prepared by extrusion of PLLA blended with 3 wt % of TAIC at 180 °C and then irradiated at 50 kGy. The irradiated tube expanded two times at 180 °C and its shape was kept at room temperature. After that, the expanded tube shrunk up to the original size by re-heating, thus could bundle wire by heat shrinking. The result is shown in Figure 7A. In another attempt, PLLA with 3 wt % of TAIC was molded to cup and plate by the extruder and irradiated to form a crosslinked structure at 50 kGy. Boiling water was poured into unirradiated and irradiated cups. The unirradiated cup deformed and changed to milky-like transparency, but the crosslinked cup kept its original shape and transparency due to protection from the crystallization of crosslinked structure (see Figure 7B).

As polylactide is also considered as an insulating material for different electronic applications, the influence of electron beam irradiation on electrical insulating properties of PLA has been studied [99]. Thus, to have better material for electric wire sheaths, a soft-resin as a plasticizer was added to polylactide but then the electric breakdown strength (E_B_) decreased. To keep the E_B_ at the same level as that of neat PLA, the composite was irradiated by the electron beam at the dose 100 kGy what resulted in the PLA crosslinking.

Examples of the use of crosslinked by irradiation polylactide in the production of packaging can be found in both the scientific literature and patents [42,100,101,102,103]. 

Thus, new films based on PLA and montmorillonite with improved barrier and mechanical properties have been developed [42]. These were designed for use with foods being processed with electron beam technology for a shelf-life extension, phytosanitary treatment, and pathogen elimination. Only low radiation doses were applied, i.e., nanocomposite films were prepared at 1, 3, and 5 wt % of clay and exposed to target electron beam doses of 1 and 10 kGy. It was observed that PLA properties were influenced by the addition of clay and by electron beam irradiation treatment: the samples showed some surface irregularities, increases of T_g_ and Young modulus, and a decrease of oxygen permeability. This limited permeability was attributed to the presence of clay and crosslinks in PLA material.

The possible application of polylactide for the production of packaging made from PLA combined with another material, e.g., cardboard is reflected in some cited in this review patents [100,101,102,103]. PLA crosslinked by gamma rays or electron beam may also find the application as adhesives in conventional glue guns [104].

Contrary to attempts of PLA crosslinking by high energy radiation, PLA photo-crosslinking has been performed on the purpose of obtaining materials for biomedicine. Among them, elastic and stiff scaffolds were prepared as well as gels which could be applied as drug delivery systems.

Amsden and his group worked for a long time on biodegradable elastomers for biomedical applications, among them polylactide-based materials make up the majority. These materials were prepared predominantly from acrylated star PLLA-PCL but also from star PLLA-poly(trimethylene carbonate) copolymers which were crosslinked by UV light. Because the main purpose of the biodegradable elastomers synthesis was their application as implants capable of releasing biologically active compounds, most studies included the preparation of elastomeric devices containing a variety of proteins (growth factors, interferons), corticosteroids, peptides [61,64,65,66,88,89,90,93,98,105]. These devices were prepared by embedding the drug (together with accompanying/solubilizing compounds) in prepolymers in bulk or solution (containing photoinitiator) in a form and irradiation with UV light. Studies included the investigation of active compounds release.

**Figure 8:**
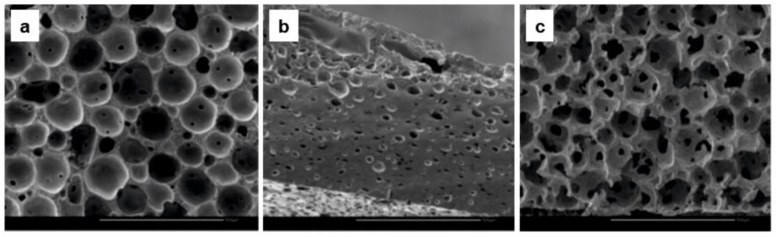
SEM images of preparation methods on elastomer scaffold structure: (**a**) scaffold made using only paraffin microbeads, (**b**) scaffold prepared using only water emulsified in the polymer solution, (**c**) scaffold prepared with combined emulsion and paraffin microbeads [91]. (Adapted with permission from Elsevier, 2009).

Another group of works concerned the preparation of elastic porous scaffolds which could be applied for the cell culture but also for the release of biological compounds [62,63,91,95,96,97]. Initially fabricated porous scaffolds (including those by Amsden group) were prepared using porogens, e.g., paraffin beads or water, for the pores generation. Among these works, the preparation of porous scaffold from 3-arm acrylated PLA/ε-CL copolymer with dual porosity (due to the introduction of two porogens, i.e., paraffin beads and water) and pore interconnectivity is situated [91] (see Figure 8**).**

As another interesting example, the preparation of the combined system, i.e., elastomeric scaffold with the mechanical strength and a hydrophilic cell encapsulating hydrogel which formed a bi-continuous two-phase cell delivery device for the repair and/or replacement of load-bearing soft tissues can be presented [95]. Thus, an elastomer from a star-poly(ε-CL-co-d,l-lactide) triacrylate (CL:DL-LA = 0.5:0.5, M_w_ = 4000 and 8000) and an N-methacrylate glycol chitosan (MGC) hydrogel to distribute the cells from bovine articular cartilage and enable cells growth has been prepared. Functionalized chitosan containing cell culture was mechanically mixed with functionalized PCL-PDLA copolymer and after the addition of photoinitiator, this material was cured in the appropriate form, using UV light. The obtained scaffolds of bi-continuous morphology had mechanical properties resembling those of soft tissues. Cell culture experiments conducted with the selected scaffold demonstrated that the chondrocytes remained viable throughout the entire manufacturing process and were able to proliferate. The authors claimed the feasibility of the scaffolds as an injectable and in situ crosslinkable cell delivery system. 

**Figure 9:**
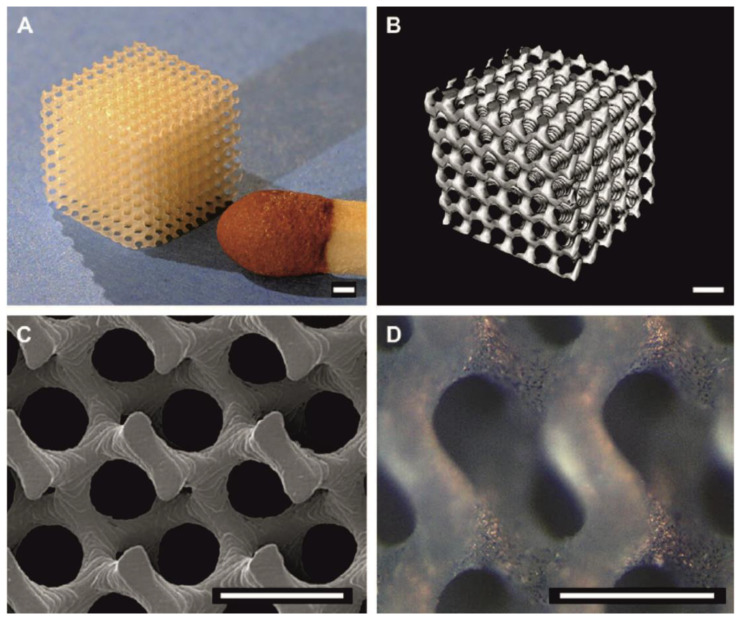
Images of PDLLA network scaffolds with a gyroid architecture prepared by stereolithography: (**A**) photograph, (**B**) microcomputed tomography (µCT) visualization and (**C**) SEM image. In (**D**) a light microscopy image is shown for a scaffold seeded with mouse pre-osteoblasts after 1 d of culturing. Scale bars represent 500 µm [57]. (With permission from Elsevier, 2009).

Many articles concern precisely designed porous tissue scaffolds prepared using stereolithography. Stereolithography is an additive fabrication process that uses a liquid light-curable photopolymer and a laser to create three-dimensional (3-D) structures [106]. Thus, porous PLA scaffolds with gyroid morphology have been fabricated using stereolithography, by visible light crosslinking of PLA macromers [57]. Complex structures could be built by illuminating sequential layers of a polymerizable resin using digital pixel masks or arrays of mirrors. In stereolithography, the thickness of a solidified layer is controlled by the light irradiation dose. It was possible to form relatively large structures (up to 42 × 33 × 200 mm) at high resolutions. The size of the smallest features that can be built was determined by the size of the light pixels (32 × 32 µm in the x and y directions), the layer thickness (25 µm), and the over cure. Although cell seeding of porous structures prepared from hydrophobic polymers, such as PDLLA is difficult, the very open structure of the gyroid architecture facilitated the penetration of water into PDLLA scaffolds prepared by stereolithography and enabled the cell seeding of mouse pre-osteoblasts. The achieved results are shown in Figure 9.

**Figure 10:**
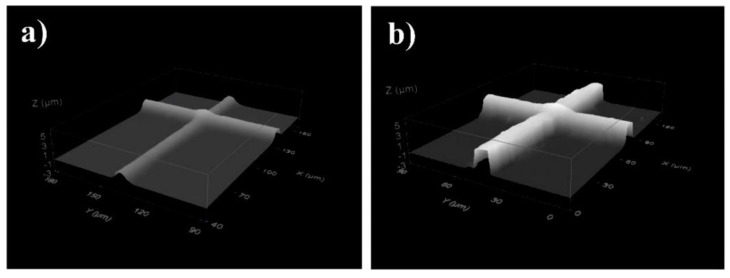
Topography images of crossing lines generated by direct laser writing using a formulation comprising the macromonomer: (**a**) linear-YNE and (**b**) star-YNE PLAs, both with a stoichiometric amount of the thiol (stoichiometry alkyne/thiol 1:2) and 3 wt % of photoinitiator. Images were obtained using a confocal microscope [107]. (Adapted with permission from Elsevier, 2017).

Photolithography was also applied for the network synthesized by a different approach [107]. PDLA network was prepared by a thiol-yne photo click reaction where alkyne functionalized star-shaped and linear PLAs were coupled with tetrafunctional thiols. Crosslinking was performed by UV irradiation of prepared polymer films. Amorphous crosslinked polymers were stable when hydrolyzed—no significant weight loss was observed during the first 10 weeks (around 4%). Films prepared by the casting of solutions containing functionalized PLA, tetrathiol and photoinitiator were also crosslinked using the direct laser writing (DLW) technique which enabled the preparation of structured patterns. Patterned samples were prepared by moving the thin film of the photopolymerizable material within the focal plane using a computer-controlled XY translation stage. Photopolymerization selectively took place in the exposed areas leaving the non-exposed material unreacted. This unexposed material was subsequently etched away using acetone as a solvent. The results are shown in Figure 10. Patterned films were used for the cell culture and independent experiments concerning cell viability indicated that studied materials based on crosslinked PLA were not toxic. The presented study showed that advanced photolithographic techniques allowed the microfabrication of well-defined micrometer-scale structures for cell patterning.

**Figure 11:**
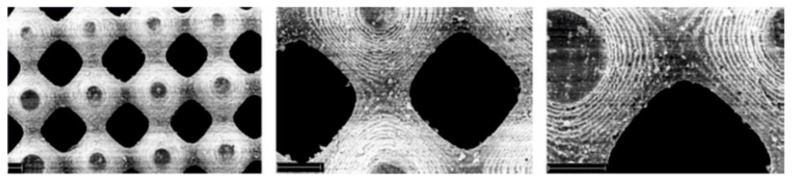
SEM images of porous structures with Schwarz primitive pore network architecture prepared by stereolithography from PDLLA and nano-HAP composite resins containing 5 wt % nano-HAP. Scale bars 200 µm [108]. (Adapted with permission from Elsevier, 2013).

Micro-stereolithography has been also used for PLA composites. For example, a well-defined three-dimensional 3D pore network has been prepared starting from composite PDLLA/nanosized hydroxyapatite (HAP) [108]. The authors dispersed nano-HAP powder in a photo-curable PDLLA macromer in N-methyl pyrrolidone (as not reactive diluent) and after the addition of photoinitiator and some additives (inhibitor and dye improving the depth of light penetration), the composition was used to fabricate porous structure in a standard stereolithography apparatus. Subsequent layers were cured a dozen times by UV irradiation. As a result, a Schwarz pore network containing 5 wt % of nano-HAP has been fabricated what is illustrated in Figure 11. The ceramic component remained well dispersed in the polymeric matrix and HAP particles on the pore surface could allow the interaction between the bone-forming nano-HAP and cells. Investigation of mechanical properties showed that with increasing nano-HAP content the elasticity modulus of the composite PDLLA/nano-HAP network materials increased.

**Figure 12:**
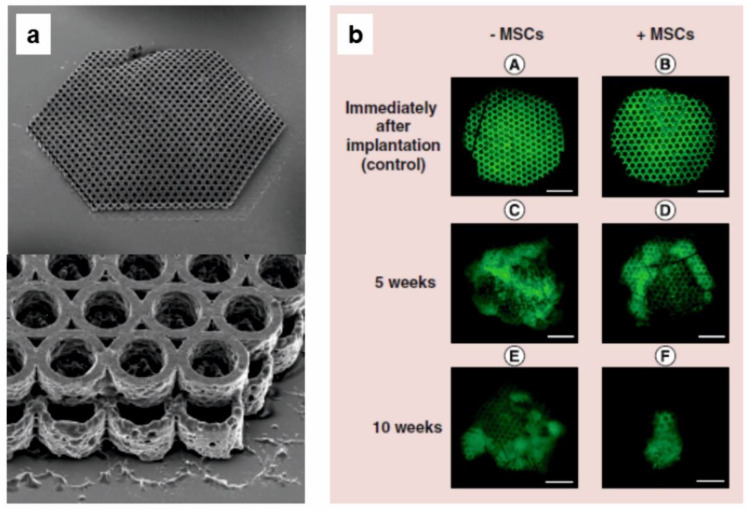
(**a**) Micrographs of a 2PP-fabricated PLA scaffold, (**b**) fluorescence of PLA scaffolds after implantation into mice; MSC—mesenchymal stem cell [71]. (Adapted from Future Medicine, 2016).

Some authors applied a two-photon polymerization technique (2PP) as the type of stereolithography [109] for the preparation of UV crosslinked PLA-based materials designed for tissue scaffolds [70,71,73,74,75]. 2PP is a computer-aided microfabrication method by which it is possible to produce biomimetic synthetic scaffolds with high precision and reproducibility. This process uses simultaneous absorption of two photons of near-infrared (780 nm) or green (515 nm) laser light. For example, photoactive material was prepared by dissolving star-shaped methacrylate-functionalized poly(d,l-lactide) (M_n_ = 2600) in dichloromethane and mixing it with photoinitiator [71]. This material was next used for the fabrication of 3D structures (shown in Figure 12a) by the 2PP technique. It was demonstrated that the fabricated PLA-based scaffolds were a beneficial microenvironment for the osteogenic differentiation of mesenchymal stem cells in vitro and the potential of prepared scaffolds as implants in cranial defects was proved by tests in vivo upon their implantation into the cranial bone defect in mice. Figure 12 illustrates prepared PLA scaffolds and their behavior as implants in mice.

The above-presented selected examples of the formation of complicated 3D structures by different stereolithography techniques concern mainly the fabrication of porous scaffolds which, as authors claim, could be used as implants for bone regeneration. However, it seems that these methods may be also useful in the production of precise elements for some other applications, as, e.g., electronic devices.

## 5. Conclusions

Different types of radiation were successfully used to crosslink polylactide which appeared very sensitive to irradiation mainly due to the presence of methine hydrogen atoms along the polymer chain as well as the possibility of introducing functional end-groups. However, not only crosslinking but also other reaction as branching and chain scission proceeds upon irradiation. The proportions of these processes depend on many factors and the specified goal can be achieved by manipulating the irradiation conditions, by PLA functionalization and by the application of reactive additives. The choice of the irradiation type and its parameters depends on these specified goals.

Polylactide-based networks with high gel fraction were synthesized from both high molecular weight PLA and its copolymers as well as from low molecular weight PLAs functionalized at the chain ends by chain linking (coupling) methods. 

The first of these approaches was mainly used in crosslinking by irradiation with high energy rays (electron beam and γ-rays). However, to have any control over crosslinking under irradiation, which can generate many radicals, multifunctional crosslinking agents containing unsaturated groups have to be added. As crosslinking by electron beam and gamma irradiation is always accompanied by chain scission, this method cannot be applied for all applications. Chain scission may be limited by adjusting the irradiation dose. The evident advantage of ionizing irradiation is the possibility to perform crosslinking at low temperatures and excellent penetration. One should, however, have in mind that the radiation affects many characteristics of the material, i.e., causes the decrease of glass transition, cold crystallization, and melting temperatures.

Crosslinking by exposure to less energetic UV (or visible) light undergoes in a more controlled manner, although undesired radical reactions may also proceed. Photo-curing is considered as the best method for crosslinking of PLA-based polymers designed for tissue engineering. For crosslinking by UV light, medium molecular weight polylactides (linear and star) functionalized with unsaturated end groups should be prepared. Polylactide macromers are mixed with photosensitive compounds being a source of radicals that initiate polymerization of unsaturated groups. Among different photo-initiators, the compounds which are reported to be not harmful in the context of biomedical applications can be chosen [110]. Because prepolymers used for crosslinking are characterized by low viscosity, it is often possible to mix polymer with the photoactive compound in bulk without using a solvent. This, in turn, enables in-situ crosslinking at low temperature, e.g., after placing liquid oligomers in a body. The weakness of this crosslinking method is the necessity of efficient PLA (meth)acrylation or another functionalization introducing reactive end groups. Moreover, radical chain-growth polymerization generates non-biodegradable high molecular weight acrylic chains. These acrylic chains become the major drawback as they are difficult to eliminate from the body. To avoid acrylic chains some authors prepared polylactide networks from prepolymers which were linked via different “click” type reactions; for biomedical application photo-induced, “thiol-ene” addition is well suited. Although photo-crosslinking seems to be a very suitable method for biomedical PLA applications, it also has a limitation, namely limited depth of penetration.

The methods presented here of radiation and photo-induced crosslinking of PLA-based materials could find an application at present (and possibly in the future) in different areas. The still developing sophisticated techniques in tissue engineering (e.g., photolithographic techniques) and in complicated drug delivery systems but also predictions for PLA mass production of durable bioplastics suggest also development of crosslinking methods for this biocompatible and bioderived polyester.

## Figures and Tables

**Table 1:** Ionizing radiation curing of polylactide.

Polylactide-Based Polymer (M_n_, g·mol^−1^)	Radiation Type, Dose	Curing Co-Agent	Gel Content, %	Achieved Results	Reference
PLLA (99,000)	Electron beam, 0–100 kGy	TAIC, TMAIC, TMPTA, TMPTMA, HDDA, derivative of EG	0.1–88	Together with annealing improved heat stability above T_g_ until T_m_; lower solubility in any solvents; retarded enzymatic degradation.	[30]
PLLA	Electron beam, 0–50 kGy	TAIC, TMAIC, TMPTA, TMPTMA, HDDA, derivative of EG	10–83	Stability at higher melting temperature; application of the crosslinked PLLA on heat-shrinkable tubes, cups and plates.	[31]
PLLA (115,100), PDLLA (197,000)	Electron beam, 0–50 kGy	TAIC	~40–100	Shifts of T_cc_ to higher and T_m_ to lower temperatures; increase in tensile strength, young’s modulus and decrease in elongation at break; the crosslinked PLA samples were harder and more brittle at low temperature, but rubbery and soft, then stable at higher temperature (over T_m_); decreased rate of enzymatic hydrolysis.	[32]
Equimolar blend of PLLA and PDLA	Electron beam, 0–50 kGy	TAIC + supercritical CO_2_	~30–90	Shift of T_m_ of homo crystals to lower temp.; improved toughness and tensile strength.	[33]
PLLA (155,500)	Electron beam 0–90 kGy	TAIC	NA	Pristine PLA: Only degradation was observed; PLA/TAIC: Increase of T_g_ (69–75 °C), decrease of melt flow and water vapor permeability.	[35]
PLA (155,500)	Electron beam, 200–1000 kGy	TAIC	68.2–89.4	For neat PLA: only degradation; PLA/TAIC: decrease of gel content with increasing radiation dose; optimum crosslinking obtained at radiation dose of 40–200 kGy and 3–5 wt % of TAIC.	[25]
PLA (155,500) /PCL (82,500) blend	Electron beam, 0–90 kGy	TAIC	NA	PLA: Increase of flexural modulus, tensile strength, flexural strength, decrease of elongation at break; PLA/PCL blend: partial degradation of PLA phase, mechanical properties depending on ratios of the polymeric components.	[34]
PLA (91,000)/PBAT (35,000) blend	Electron beam, 0–90 kGy	TAIC	40–90	Crosslinking and degradation after irradiation mostly in PLA phase, PBAT less susceptible to radiation influence.	[39,40]
PLLA + Reinforced by flax fiber (20 wt %)	Electron beam, 0–40kGy	TAIC	7.6–62.5	Increase of tensile strength of about 20% in the presence of TAIC at 40kGy of irradiation dose; irradiation in the presence of TAIC led to reduced enzymatic degradation; decrease of interfacial adhesion of flax fibers and PLA matrix in the presence of TAIC.	[41]
PLA (210,000) + MMT (1,3,5 wt %)	Electron beam, 1 and 10 kGy	-	NA	Increase of T_g_, crystallinity and young modulus, decrease of elongation at brake and oxygen permeability.	[42]
PLA/PEGM/HBN blend composite	Electron beam, 0–100 kGy	-	NA	At low doses: partial branching and crosslinking for neat PLA and PLA/PEGM; at higher doses: chain scission dominates. increase of T_g_, notched impact strength and heat deflection temp. with radiation of blend-composites with higher amount of HBN; accelerated hydrolytic degradation of irradiated blend and blend-composites.	[43]
PLLA	γ-rays 2.5–50 kGy	TAIC	10–100	Decrease of swelling with increasing gel content, decrease in elongation (75%), maintenance of tensile strength, decrease of crystallinity (from 36 to 10%) and T_m_ (from 182 to 165 °C).	[36]
PLA + Flax fiber (5 wt %)	γ-rays 0–20 kGy	TAIC	70–90	Increase of the gel fraction in PLA/ flax composite with the radiation dose, degradation at higher doses; improvement of tensile strength and toughness with the increase in the radiation dose, decrease of elongation at break.	[37]
PLA (106,000)	γ-rays 0–100 kGy	TAIC, Ov-POSS	Up to 80	Higher degree of crosslinking for PLA/OvPOSS in comparison to PLA/TAIC; irradiated composites exhibited decrease of crystallinity, lower elongation at break and higher E-modulus, higher thermal stability and heat deflection temp. than that of neat PLA	[44]
PLA (72,000)	γ-rays 0–20 kGy	TAIC as crosslinking agent (CA), Epoxy functional acrylic oligomer (Joncryl^®^ ADR 4368) as chain extender (CE)	1.2–46.2	Considerable gel formation was observed for PLA/CA at high irradiation dose; addition of CA or CE increased the shear viscosity of neat and irradiated PLA; addition of CA and CE enhanced T_c_ and decreased crystallinity; improvement of tensile properties was higher for CA.	[38]

TAIC—tiallyl isocyanurate; TMAIC—trimethylallyl isocyanurate; TMPTA—trimethylolpropane triacrylate; TMPTMA—trimethylolpropane trimethacrylate; HDDA—1,6-hexanediol diacrylate; EG—ethylene glycol bis(pentakis(glycidyl allyl ether))ether, hydroxy terminated; PDLLA—polylactide prepared from racemic mixture of D-LA and L-LA; PBAT—poly(butylene adipate-co-terepthalate); PEGM—poly(ethylene-co-glycidyl methacrylate); HBN—hexagonal boron nitride; Ov-POSS—octavinyl polyhedral oligomeric silsesquioxane; MMT—montmorillonite; NA—not available.

**Table 2:** Conditions of photo-crosslinking of linear and star PLA low molecular weight (co)polymers and observed results. UV light was used to induce crosslinking (visible or laser light/2PP, when indicated).

PLA Structure (M_n_, g·mol^−1^)	Crosslinking Group	Photoinitiator	Gel Content ^a^, %	Achieved Results	Ref.
PDLLA-*b*-PEG-*b*-PDLLA (1000–20,000)	Acrylate end group	2,2-dimethoxy-2-phenylacetophenone (Irgacure 651)	65–74	Degradation rate increased with increasing M_n_ of precursor; materials used in the sustained release of proteins.	[48]
PDLLA-*b*-PEG-*b*-PDLLA Or: P(DLLA-*co-*TMC)-*b*-PEG-*b*-P(DLLA-*co-*TMC (4500–5500)	Fumarate end group	2,2-dimethoxy-2-phenylacetophenone	>90	Hydrogels prepared in N-vinylpyrrolidone were used for the study of model protein release; the degradation behavior could be controlled by changing the composition of the hydrophobic segments.	[49]
PDLLA-*b*-PEG-*b*-PDLLA (~1600)	Acrylate end group	2,2-dimethoxy-2-phenyl acetophenone		Preparation of porous scaffolds for the study of the growth factor encapsulation and release and implantation in the case of cranial defect.	[50]
PDLLA-*b*-PEG-*b*-PDLLA (990–1240)	Acrylate end group	camphorquinone/ethyl-4-*N,N*-dimethylaminobenzoate	89–100	Modification of hydrophobicity (contact angle 123°–142°); T_g_ = 1.8–26 °C depending on the composition and crosslinking density; tensile modulus in the range 0.92–3.67 MPa and strain at break 0.19–0.65; preparation of scaffolds with various pore sizes by salt-leaching method.	[52]
PDLLA-*b*-PEG-*b*-PDLLA (1120–10,720)	Acrylate end group	2,2-dimethoxy-2-phenylacetophenone	78–100	Both lower crosslinking density (higher M_n_ of macromer) and the lower crystallinity (lower M_n_) increased the degradation rate of the networks; the maximum improvement in penetration force, lubricant property, over control was 41% in the needle coated with PPG-based polymer network.	[53]
PDLLA-*b*-PPG-*b*-PDLLA (1150–4720)	Acrylate end group	2,2-dimethoxy-2-phenylacetophenone	93–99		
PDLLA-*b*-PTMG-*b*-PDLLA (1370–3620)	Acrylate end group	2,2-dimethoxy-2-phenylacetophenone	95–97		
PDLLA-*b*-PCL (1570–2390)	Methacrylate end group	camphorquinone/ethyl-4-dimethylaminobenzoate	Highly crosslinked	Decrease of T_g_ with increasing CL content (T_g_ in the range −30 to 60 °C). Storage moduli in the glassy regime were similar, in the rubbery regime dependent on crosslinking density; highly cross-linked scaffolds were cellularly compatible and promoted osteoblast attachment.	[51]
P(CL-*co*-LLA-*co*-GA) (1870–10,190)	Acrylate end group	2,2-dimethoxy-2-phenylacetophenone	>95	Increase of T_g_ of 2.8–14.9 °C, similar ultimate strength (σ = 2.39–3.76 MPa); Young’s modulus (E = 1.66–12.29 MPa and maximum strain (ε = 21–176%); Excellent biocompatibility of films with smooth muscle cells.	[54]
P(LDLA-co-GA)-b-PEG-b- P(LDLA-co-GA) (~5300)	Itaconic end groups	camphorquinone	94–98 ^b^	Swelling properties depended on crosslinking time, thus crosslinking density; with longer UV exposure better hydrolytic stability of hydrogel was observed.	[55]
P(DLLA-*co*-TMC) (27,000–29,000)	Methacrylate end group	Irgacure 2959	74–90	Depending on the DLLA /TMC ratio, amorphous networks with T_g_ of 13 to 51 °C and elastic modulus from 3.6 MPa to 2.7 GPa were obtained; networks of more than 40 mol% of TMC are tough, flexible and elastomeric at r.t. with elongations at break of up to 800%. When DLLA:TMC = 60:40, T_g_ is between 25 and 37 °C, thus elastic medical devices with SM properties could be implanted in a temporary shape.	[56]
2,3- and 6-arm PDLLA (6600–34,200)	Methacrylate end group	2-hydroxy-1-[4- (hydroxyethoxy)phenyl]-2-methyl-1-propanone (Irgacure 2959)	96	T_g_ (55–76 °C) dependent on macromer chain length; mechanical properties similar to HMW PDLLA- suitable for stereolithography; mouse pre-osteoblasts readily adhered and proliferated well on networks.	[57]
3-arm P(TMC-*co*-DLLA) (3100–4000)	Fumaric acid monoethyl ester	2,2-dimethoxy-2-phenylacetophenone	67–81^c^	The E modulus decrease with TMC content, tensile strength and elongation at break unaffected. Relative low values of tensile strength (1–2 MPa), and E modulus (1–10 MPa) in comparison with HMW PDLLA and PTMC.	[58]
4-arm PDLLA-*co*-PCL (5000–10,000)	Acrylate end group	1-hydroxycyclohexylphenylketone (irgacure 184)	NA	Fabrication of microstructures by soft lithography. Possibility of using studied materials to culture mammalian cells.	[59]
4-arm P(LLA-b-CL) (Mn ~3200–12,000)	Methacrylate end group	Camphorquinone ^d^	NA	Transition temperatures depended on the length of poly-CL segments. Decrease of T_m_ and crystallinity with increasing M_n_. Thermo-responsive properties as permeability of a drug.	[60]
3-arm P(CL-*co*-DLLA) (1250–7800)	Acrylate end group	2,2-dimethoxy-2-phenylacetophenone	>95	T_g_ of elastomers below physiological temperature (even below 0 °C). The Young’s modulus and stress at break inversely proportional but strain at break-proportional to the prepolymer M_n_. The ability of elastomeric devices to encapsulate (glyco)proteins and release them according to an osmotic pressure delivery mechanism; confirmed ability to degradation in vitro and in vivo. Preparation of porous scaffolds capable to degradation with mechanical properties dependent on prepolymers M_n_. Ability to adsorb proteins and to cell proliferation; dependence of adsorbed protein layer on the material stiffness.	[87,88,89,90,91,92]
3-arm poly(CL-co- DLLA) (1250, 2700 and 3900)	Acrylate end group and co-photo-crosslinker poly(ethylene glycol)diacrylate (PEGDA) (4000 and 24,000)	2,2-dimethoxy-2-phenylacetophenone	95–98	T_g_, T_m_ and ∆H_f_ varied with prepolymer M_n_, co-photo-crosslinker amount and M_n_. Networks without PEGDA were amorphous, with PEGDA indicated melting; preparation of cylindrical elastomeric devices able to encapsulate Vitamin B_12_.	[64]
3-arm poly(TMC-co-DLLA) (7800–8500)	Acrylate end group	2,2-dimethoxy-2-phenylacetophenone	79–88	With increasing amount of DLLA increase of Young’s, stress at break, T_g_ and decrease of elongation at break. The possibility of osmotic pressure driven release of proteins. Study of the behavior of elastomers implanted into rats.	[93,94]
3-arm poly(TMC-DLLA-CL) (2300–7800)	Acrylate end group and co-photo-crosslinker poly(ethylene glycol)diacrylate (PEGDA)	2,2-dimethoxy-2-phenylacetophenone	86–99	T_g_ (−18 to 2 °C) varied with the monomer composition and the M_n_ of PEGDA. Preparation of cylindrical elastomeric devices able to swell and to encapsulate corticosteroid and growth factors utilizing the osmotic pressure mechanism.	[65,66,67]
Poly(LLA-co- CL-acryolyl carbonate) (17,900–22,600)	Pendant acrylate group	2,2-dimethoxy-2-phenylacetophenone	90	Preparation of fibrous scaffolds by melt electrospinning writing; Stiffness of the scaffolds increased significantly (up to ∼10-fold) after crosslinking with UV compared with un-crosslinked scaffolds; the preservation of stiffness upon repetitive loading.	[62]
Poly(L-lactide-co-acryolyl carbonate) (55,900–72,100)	Pendant acrylate group	NA	84–94	Increase of T_g_, decrease of T_m_ and degree of crystallinity after crosslinking; Electrospun and photo-crosslinked polymer resulted in scaffolds with increased tensile modulus in comparison with uncrosslinked fibrous scaffolds; good cytocompatibility toward fibroblasts of crimp-stabilized scaffolds.	[63]
3-arm Poly(DLLA-co-CL) (M_w_ 4800–10,900)	Acrylate end group and co-photo-crosslinker N-methacrylated glycol chitosan (MGC)	Irgacure 2959	98–100	Preparation of bi-continuous two-phase (elastomer /hydrogel) cell delivery device for the repair and/or replacement of load-bearing soft tissues. Decrease of elastic modulus with increasing content of MGC; using electrospinning for scaffold preparation.	[95,96,97]
3-arm Poly(DLLA-co-CL) (2700 and 5000)	Acrylate end group and co-photo-crosslinker diacrylate oligo(d,l-lactide)-b-poly(ethylene glycol)-b-oligo(d,l-lactide)	2,2-dimethoxy-2-phenylacetophenone	>95	Enhancing the degradation rate by introducing PEG fragment; regulation of the degradation rate and peptide release by M_n_ of PEG and M_n_ of prepolymer.	[98]
4-arm PDLLA (1500–9500)	Methacrylate end group (methacrylic anhydride or 2-isocyanatoethyl methacrylate)	2,2-dimethoxy-2-phenylacetophenone	90–99	Increasing of T_g_ with decreasing M_n_ of precursors; networks based on low M_n_ oligomers were generally more rigid, those based on high M_n_ exhibited higher elongation; mechanical properties differ with type of precursors methacrylate end group.	[68]
PDLLA (1310) + TEGDMA as reactive diluent + Hydroxyapatite (HA) as bioactive filler	Methacrylate end group	Camphorquinone/ N,N^’^-dimethylaminoethyl Methacrylate	77–100	T_g_ (38–55 °C), flexural strength (3.5–94 MPa) and flexural modulus (75-3980 MPa) were dependent on composition of polymer resin and an amount of HA; increasing thermal stability with increasing amount of filler. Higher gel content and higher concentration of HA led to decreased rate of degradation; higher HA content resulted in the less cytotoxic sample.	[69]
4-arm Poly(d,l-lactide) (2600 or 2400 or 450–820)	Methacrylate end group	4,4′-bis(dimethylamino)benzophenone ^e^	NA	Preparation of scaffolds with Young’s modulus even bigger than 4 GPa for the mesenchymal stem cells osteogenic differentiation; Independently—collagen reinforcement: about one order of magnitude increased Young’s modulus for the hybrid matrix without affecting its cytotoxicity;	[72,73,74,75]
4-arm Poly(L-lactide) (M_w_ 1250)	Methacrylate end group	Irgacure 369 ^e^	NA	Preparation of scaffolds for supporting Schwann cell growth—neural scaffolds in nerve repair.	[70]
Poly(LLA-co-GMA) (1650–3260)	Pendant methacrylate group	Camphorquinone/ N,N′-dimethylaminoethyl methacrylate	72–95	With increasing content of GMA (9.5–19.2 mol%) the increase of gel content, compressive stress (3–25.5 MPa) and the decrease of degree of swelling was observed; Increase of T_g_ by 15–20 °C in comparison with original copolymer.	[76,77]
PLLA (M_V_ 276,500)	-	Benzophenone	38–98.5	Slight increase of T_g_ in comparison with pristine PLA, decrease of T_m_ and crystallinity; improvement of thermal stability; with increase of gel fraction—increase of storage modulus (from 5.4 to 9.6 GPa at 0 °C), tensile strength (from 48 to 81 MPa), modulus (from 1.8 to 3.1 GPa), toughness (from 67 to 82 MPa) and decrease of strain (from 3.9 to 1.6%).	[78]
PLLA - diacyl of 5-cinnamoyloxyisophthalic acid (ICA) (10,000–34,500)	Pendant 3-phenylprop-2-ene group	-	50–100	Slight increase of T_g_ (from 50 to 53 °C), decrease of crystallinity (from 10 to 3 %), slight decreases of T_m_, thermal decomposition T_d_, increase of ultimate tensile strength (from 13 to 23 MPa), decrease in elongation (from 12 to 5.2%), increase of Young’s modulus E (from 483 to 830 MPa); Decrease of degradation rate.	[82]
PLLA- diacyl of 4,4′-(adipoyldioxy)dicinnamic acid (8700–43,500) ^f^	Main-chain 3-phenylprop-2-ene group	-	9–86	Increase of T_g_ (from 51 to 53 °C); decrease of ∆H_m_ (from 4.8 to 0.1 J.g^−1^), small decrease of T_m_ (from 150 to 147 °C), increase of thermal decomposition T_d_; increase of tensile strength and tensile modulus and decrease of elongation at break with increasing photocuring time and gel content; decrease of degradation rate.	[83]
P(LLA-co-MC) (12,900–65,100) ^f^	Pendant phenylprop-2-ene group	-	NA	The kinetic of UV crosslinking was studied by FT IR spectroscopy.	[84]
PLA_50-_Pluronic^®^-PLA_50_ (50,000-200,000)	-C-H- bond in polymer chain	Aryl-azide group	Up to 55	Preparation of elastic microfibers (elastic limit–*ε_y_* up to 182 %) for soft tissues by electrospinning.	[85]

^a^ Gel content was dependent on M_n_ and/or copolymer composition; ^b^ determined by conversion of double bonds; ^c^ gel content was dependent on increasing UV energy and photoinitiator concentration^; d^ visible light; ^e^ two-photon polymerization technique (2PP); ^f^ M_n_ of the polycondensation product; PPG—poly(propylene glycol); PTMG—poly(tretramethylene glycol); TMC – 1,3-methylene carbonate (1,3-dioxan-2-one); TEGDMA—triethylene glycol dimethacrylate; MC—5-methyl-5-cinnamoyloxymethyl-1,3-dioxan-2-one; GMA—glycidyl methacrylate; NA—data not available.
